# Correction to: ‘Imputation of body mass’ (2021) by Turvey *et al.*

**DOI:** 10.1098/rspb.2026.2193

**Published:** 2026-10-28

**Authors:** Samuel T. Turvey, Clare Duncan, Nathan Upham, Xavier A. Harrison, Liliana M. Dávalos

**Affiliations:** ^1^ Institute of Zoology, Zoological Society of London, London, UK; ^2^ School of Life Sciences, Arizona State University, Tempe, AZ, USA; ^3^ Department of Biosciences, University of Exeter, Penryn, UK; ^4^ Department of Ecology and Evolution, Stony Brook University, New York, USA; ^5^ Consortium for Inter-Disciplinary Environmental Research, Stony Brook University, New York, USA

We previously regressed the survival of non-volant Caribbean Holocene mammals as a function of body mass and island characteristics [1]; body mass was lacking for one species, and island covariates were missing for several islands. We believed covariate values had been imputed, but we were wrong as we will explain in this correction. Several approaches have been developed to impute missing covariates in hierarchical Bayesian modelling and all involve specifying a sub-model to impute the missing covariate based on, for example, geography [2], or another covariate [3]. In the hierarchical Bayesian approach that we previously used [1], integrated nested Laplacian approximations (INLAs), the posterior predictive distribution can be used to impute values of the response variable [3] (in our case, binary survival or extinction in the logit scale). But imputing covariates requires specifying appropriate sub-models based on sources of information that relate to the covariate. We conflated one approach—the posterior prediction of the response—with covariate imputation. Consequently, the covariate values we attempted to impute were incorrect [1]. As the results from incorrectly imputed covariates were reported, those could be wrong too. We therefore applied the correct method of imputation for the critical covariate, body mass, to our data. After undertaking those analyses, all key conclusions of our paper hold [1].

There were missing covariates for both body mass and island characteristics, but we lack a sound basis to impute island values. Of 118 islands, 87 lacked dates of first human arrival, 86 lacked hurricane metrics and 78 lacked human impact values. These missing covariates were not randomly distributed and instead clustered, with 68 islands lacking all 3 covariates. We therefore focused imputation on a phylogenetic model of body mass as a normally distributed variable,

$$\begin{array}{r} y_{i}∼\;Gaussian\left(\mu ,\sigma^{2}\right), \end{array}$$

in which μ is the mean, and *σ*^2^ is the variance. Given body mass observations per species *y_i_*

$$\begin{array}{r} \;y_{i}=\;\beta_{0}+\beta_{1}+\beta_{2}+\;\varepsilon_{i}, \end{array}$$

with the global intercept given by $\beta_{0}$, and non-phylogenetic species-specific intercepts given by

$$\begin{array}{r} \beta_{1}\;∼Gaussian\left(0,\;\sigma_{0}^{2}I_{species}\right), \end{array}$$

the phylogenetic variance–covariance $V_{species}$ also generates species-specific effects modelled by

$$\begin{array}{r} \beta_{2}\;∼Gaussian\left(0,\;\sigma_{0}^{2}V_{species}\right). \end{array}$$

This last set of species-specific intercepts accounts for the correlated structure of errors and non-independence in observations through the phylogeny. We implemented these models with a single phylogeny from [4] and using the R package phyr v1.1.2 [5]. Following [3], we then generated 100 approximated posterior samples from the fitted model using the *inla.posterior.sample* routine in INLA [6]. These provide a frequency distribution of body mass imputed for the one species that lacked this value: *Hexolobodontinae* sp. nov., summarized by a low mode (1.29 kg), intermediate median (2.47 kg) and a high mean (5.3 kg), indicating a long tail in the posterior sample.

As in Rayfield, Cooke [7], we then modelled the probability of survival for each species-by-island observation by looping each imputed body mass value across the sample of 100 phylogenies from [4] such that each of 100 models comprised a unique imputed body mass and phylogeny combination. These results thus propagate the error of the imputed body mass to the extinction models. We used custom scripts to summarize those new results, shown in figure 1.

**Figure 1.**
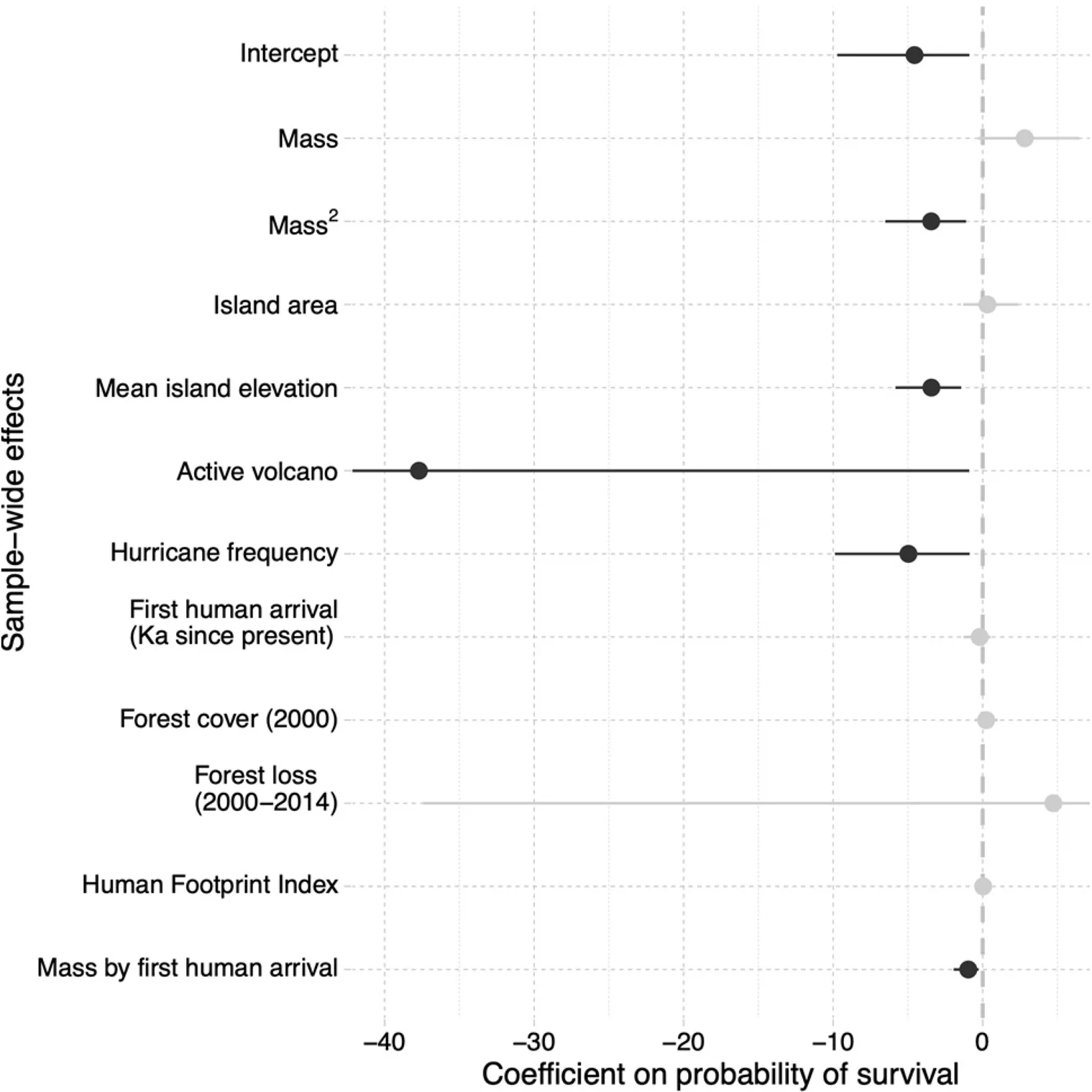
Posterior estimates and high-probability intervals for sample-wide model coefficients including all non-collinear covariates and a single imputed body mass.

Compared to published results [1], all significant covariates are confirmed (figure 1). Volcano presence, however, was originally a negative covariate of survival probability (figure 2), but now this relationship is both stronger and statistically supported. Nearly identical results were obtained in analyses without the imputed body mass value (not shown).

**Figure 2.**
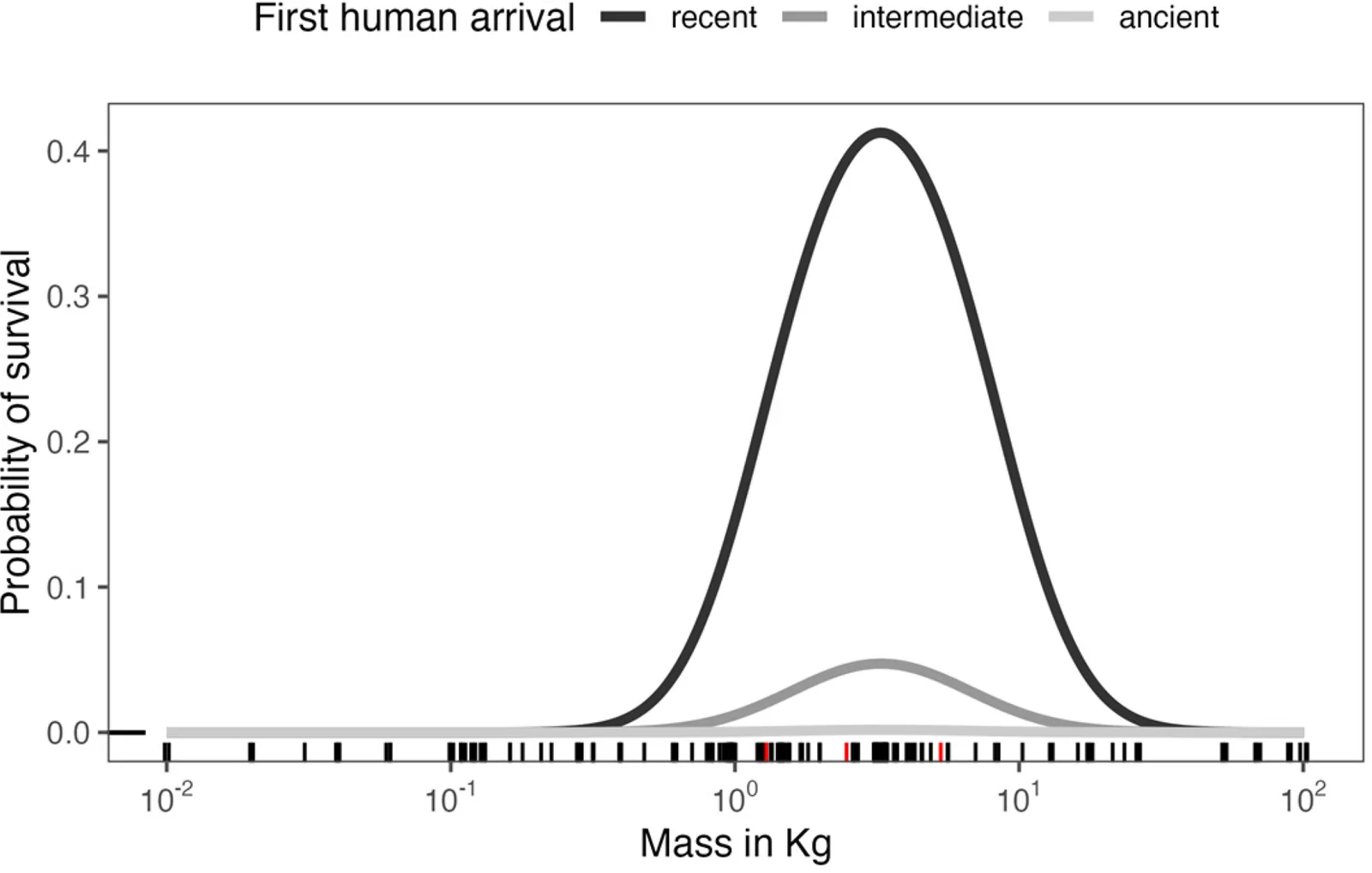
Survival probability as a function of body mass for islands at the most recent quartile (recent), median (intermediate) and top quartile (ancient) of first human arrival, with all other covariates corresponding to their sample means. Bottom ticks show jittered species body masses, with imputed body mass values (in order: mode, median, mean) in red.

To determine how the relationship between body mass and extinction probability had changed, we replotted predicted estimates from the phylogenetic regression (including the new values imputed for *Hexolobodontinae* sp. nov.). Results for these analyses were nearly identical to our original results [1], and patterns of intermediate body mass survival and interactions with first human arrivals were the same.

Results from our re-analyses, including a phylogenetic imputation sub-model for body mass, are consistent with the findings first reported in our paper [1]. As the incorrect imputations were part of our code, we have updated the GitHub repository to reflect these changes [8].

## Data Availability

Data are available on GitHub [8].

## References

[1] Turvey ST , Duncan C, Upham NS, Harrison X, Dávalos LM. 2021 Where the wild things were: intrinsic and extrinsic extinction predictors in the world's most depleted mammal fauna. Proc. R. Soc. B 288, 20 202 905. (doi:10.1098/rspb.2020.2905) PMC7944111 33715429

[2] Agarwal DK , Silander JJA, Gelfand AE, Dewar RE, Mickelson JJG. 2005 Tropical deforestation in Madagascar: analysis using hierarchical, spatially explicit, Bayesian regression models. Ecol. Model 185, 105–131. (doi:10.1016/j.ecolmodel.2004.11.023)

[3] Gomez-Rubio V , Cameletti M, Blangiardo M. 2022 Missing data analysis and imputation via latent Gaussian Markov random fields. SORT 46, 217–244. (doi:10.48550/arXiv.1912.10981)

[4] Upham NS , Esselstyn JA, Jetz W. 2019 Inferring the mammal tree: species-level sets of phylogenies for questions in ecology, evolution, and conservation. PLoS Biol. 17, e3000494. (doi:10.1371/journal.pbio.3000494) PMC6892540 31800571

[5] Li D , Dinnage R, Nell LA, Helmus MR, Ives AR. 2020 phyr: an R package for phylogenetic species-distribution modelling in ecological communities. Methods Ecol. Evol. 11, 1455–1463. (doi:10.1111/2041-210X.13471)

[6] Rue H , Martino S, Chopin N.. 2009 Approximate Bayesian inference for latent Gaussian models using integrated nested Laplace approximations (with discussion). J. R. Stat. Soc. B 71, 319–392. (doi:10.1111/j.1467-9868.2008.00700.x)

[7] Rayfield KM , Cooke SB, Mychajliw AM, Turvey ST, Upham N, Vaccaro S, Dávalos LM. 2026 Interactions between intrinsic and extrinsic factors shape island bat survival and extirpation in a global extinction hotspot. Evol. J. Linnean Soc. 5, kzag014. (doi:10.1093/evolinnean/kzag014) PMC13387144 42483542

[8] Upham N. 2026 Updating code for 2026 correction. GitHub. https://github.com/n8upham/CaribbeanExtinctions-WTWTW/tree/master/binomial

